# Factors associated with COVID-19 mortality in municipalities in the state of São Paulo (Brazil): an ecological study

**DOI:** 10.1590/0037-8682-0447-2021

**Published:** 2022-04-08

**Authors:** Rafaela Caroline de Souza, Ettore Rafael Mai Almeida, Carlos Magno Castelo Branco Fortaleza, Hélio Amante Miot

**Affiliations:** 1 Faculdade de Medicina de Botucatu, Departamento de Infectologia, Dermatologia, Diagnóstico por Imagem e Radioterapia, Botucatu, SP, Brasil.

**Keywords:** COVID-19, SARS-CoV-2, Mortality, Ecological studies, Health inequality, Social inequality, Human development index

## Abstract

**Background:**

The mortality rate of coronavirus disease (COVID-19) in the state of São Paulo is highly heterogeneous. This study investigated geographic, economic, social, and health-related factors associated with this discrepancy.

**Methods:**

An ecological study compared COVID-19 mortality rates according to geographic, economic, social, and health-related variables during initial infection of 2.5% of the population in municipalities with more than 30,000 inhabitants.

**Results:**

Mortality was positively associated with demographic density and social inequality (Gini index), and inversely associated with HDI income and longevity of these municipalities, accounting for 33.2% of the variation in mortality.

**Conclusions:**

Social determinants influenced COVID-19 outcomes.

As COVID-19 has had a devastating progression in Brazil, approaches that explore differences in incidence and mortality between municipalities are vital for identifying factors associated with the heterogeneous behavior of the disease from a population perspective.

One of the main epidemiological characteristics of COVID-19 is its high transmissibility (R0: 2-4). However, the existence of asymptomatic transmitting individuals, combined with variable incubation periods (5-12 days) and a high transmission rate, has hampered basic isolation measures and favored rapid global progression^1^.

The World Health Organization (WHO) declared COVID-19 an international public health emergency on January-30 and a pandemic on March-11, 2020. By mid-October 2021, approximately 240 million cases and nearly 4.9 million deaths were registered worldwide. However, testing failures, neglect of asymptomatic cases, pandemic denial, failure to register cases and deaths, and insufficient diagnostic test sensitivity (about 70%) raise the hypothesis that these numbers are underestimated, especially in regions of greater social vulnerability^2^ ^-^ ^4^.

The natural history of COVID-19 indicates the need for hospitalization in approximately 10%-20% of those infected, and lethality varies between 2% and 4% of cases. However, there is high variability in the epidemiological profiles of patients who progress to severe disease. Male sex, older age, obesity, comorbidities (e.g., diabetes, hypertension, and heart disease), immunosuppression, and neurological disorders are the main risk factors linked to the host, as identified in all international epidemiological series^1^ ^,^ ^5^. Furthermore, elements linked to the agent, such as more contagious variants (e.g., alpha B.1.1.7, beta B.1.351, delta B.1.617.1/2/3, and gamma P.1 strains), are associated with higher infection and mortality rates, mainly due to overload on the health system^6^.

On all continents, the highest rates of infection and mortality among black people, indigenous people, and low-income individuals warned that social determinants of health, which constitute socioeconomic, cultural, and behavioral factors, influence COVID-19 outcomes^7^ ^,^ ^8^.

An ecological study that analyzed the incidence and mortality of COVID-19 in Brazilian states identified an association between social inequality, economic factors, and disease outcomes^9^. However, Brazil is a large and heterogeneous country in which health coverage, ethnicity, geography, and social characteristics are highly regionalized. Furthermore, municipalities affected by the pandemic at different times and local policies to deal with the crisis also differed among them, which justifies a detailed study of the effect of these factors, at the municipal level, within a federative unit with an adequate case registration system, considering the municipal dimension of the epidemic.

This study aimed to explore the demographic, geographic, socioeconomic, and healthcare aspects associated with mortality from COVID-19 in the most populous cities in the state of São Paulo at similar epidemiological moments.

An ecological study examined municipalities in the state of São Paulo with a population greater than 30,000 inhabitants, as estimated by the Brazilian Institute of Geography and Statistics (IBGE) for 2020. The choice of municipalities was based on the expectation of a minimally structured health system.

The study’s main outcome was the COVID-19 mortality rate, published by the State Data Analysis System Foundation (Seade; https://www.seade.gov.br/coronavirus/), when the number of cases in each municipality had reached the equivalent of 2.5% of its population. An early pandemic approach for each municipality was chosen to minimize the influence of vaccination during the pandemic and to reduce the effects of different waves of infection during the pandemic, equalizing the situational progression among them.

Demographic data (demographic density and urban population percentage), geographic measures (mean temperature, altitude, latitude, and distance from the capital), social factors (HDI income, HDI longevity, HDI education, and the Gini index), and health information related to the municipalities (number of hospital beds and health institutions) were assessed.

The association between mortality and independent variables was explored using a generalized linear model with an identity link function, gamma probability distribution, and a robust covariance matrix. Collinearity was assessed using the Durbin-Watson indicator, and the adequacy of the model was confirmed by the normality of the residuals. The effect size was estimated using raw and standardized β coefficients (β_SE_ - to compare variables on different scales)^10^. The data were analyzed using IBM SPSS v25 software. The significance level was set at p ≤0.05^11^.

A total of 203 municipalities (33.5% of the state) with a population of greater than 30,000 inhabitants, accounting for 93.1% of the state's population, were included in the analysis.

Cases in the municipalities reached a rate of 2.5% relative to their total population during epidemiological periods that ranged from 131 to 589 days from the first case registered in the state (February-26, 2020). Overall, in the state of São Paulo, the time from the first case to infection in 2.5% of the population was 252 days.When the number of pandemic cases reached the equivalent of 2.5% of the population of each municipality, the average mortality (standard deviation) from COVID-19 was 7.07 (2.86) per 10,000 inhabitants, ranging between 1.00 and 15.50 deaths per 10,000 inhabitants. Overall, in the state of São Paulo, the mortality rate of COVID-19 (2.5% of the affected population) was 8.81 per 10,000 inhabitants.

Data regarding the demographic, geographic, socioeconomic, and healthcare variables are displayed in Table 1. There was high variability in the values of the variables among municipalities, resulting in a model that explained up to 33.2% of the variation in mortality from COVID-19. 

**TABLE 1:** Descriptive data and their association with mortality from COVID-19 (deaths / 10,000 inhabitants), regarding demographic, geographic, economic, social, and health-related variables, in municipalities with more than 30,000 inhabitants in the state of São Paulo when the number of cases reached 2.5% of the affected population (n = 203).

Variables	Descriptive				Univariable analysis			Multivariable analysis			
	Mean	SD	Minimum	Maximum	β	SE	p-value	β	SE	β_SE_	p-value
Latitude (° South)	22.60	1.05	24.70	20.03	**0.50**	**3.54**	**0.002**	**0.78**	**0.33**	**0.30**	**0.019**
Gini Index (2010)	0.49	0.05	0.38	0.69	**8.81**	**3.93**	**0.025**	**21.90**	**4.43**	**0.36**	**0.000**
HDI income (2010)	0.75	0.04	0.66	0.89	-3.64	4.28	0.395	**-30.24**	**7.80**	**-0.36**	**0.000**
HDI Longevity (2010)	0.85	0.02	0.79	0.89	**-22.52**	**11.62**	**0.050**	**-19.45**	**9.70**	**-0.13**	**0.045**
HDI Education (2010)	0.71	0.04	0.60	0.81	0.61	5.25	0.908	-1.88	6.26	0.01	0.764
Altitude (1000 m)	0.60	0.218	0.001	1.64	0.82	0.80	0.311	-1.13	1.04	-0.13	0.275
Urban Population (%)	82.61	8.61	31.37	96.64	**0.04**	**0.02**	**0.013**	0.03	0.02	0.12	0.135
Health Facilities / 10^4^ inhabitants	3.68	1.62	0.66	10.19	**-0.34**	**0.10**	**0.001**	-0.08	0.12	-0.08	0.502
Health beds / 10^4^ inhabitants	22.30	26.57	0.00	245.09	**-0.01**	**0.00**	**0.013**	0.01	0.01	0.04	0.380
Average annual temperature (°C)	21.15	2.10	14.50	28.00	**-0.30**	**0.07**	**0.000**	-0.06	0.09	-0.10	0.533
Distance from the capital (1000 km)	0.21	0.16	0.00	0.65	**-4.07**	**0.88**	**0.000**	-1.50	2.11	-0.14	0.478
Demographic density (/km²)	997.64	2264.79	15.70	1.4403.20	1.93*	0.25*	**0.000**	2.86*	0.47*	**0.52**	**0.000**

p (model) <0.001, p (constant) <0.001, R2 (standardized) = 33.2%, Durbin-Watson = 1.87. *Log transformation; **SD:** standard deviation; **HDI:** Human Development Index; **bold values:** p-value <0.05; **SE:** standard error; **βSE:** standardized beta coefficient (β * SD independent variable / SD dependent variable).

Demographic density and the Gini index were the variables with the greatest weight in the model (β_SE_ = 0.52 and 0.36), they were positively associated with mortality, as well as latitude. Mortality was negatively associated with the HDI and HDI longevity.Demographic, geographic, and socioeconomic factors accounted for up to one-third of the variation in the COVID-19 mortality rate in the most populous municipalities of São Paulo.

In addition to its large territory, São Paulo has diverse economic, social, demographic, and geo-climatic characteristics, which favor the exploration of these factors regarding mortality from COVID-19. Therefore, it is relevant to investigate the ecological elements that explain the variation in mortality between municipalities, which, at the same epidemiological points, was up to 15 times higher extreme cities.As of July 21, 2021, 3,966,009 cases and 135,973 deaths have been reported in the state, corresponding to 20.42% of the total cases and 24.99% of the total deaths in Brazil. as the state mortality rate as of that date, was 30.32 per 10,000 inhabitants.In the state of São Paulo, the pandemic started in the capital city and progressed through two patterns: contiguous and hierarchical diffusion. In contiguous diffusion, dispersion occurs from the capital’s metropolitan area to adjacent urban areas. In the hierarchical pattern, dissemination takes place over long distances through major highways to cities of regional relevance^12^. 

Our study confirmed that the municipalities evolved with different pandemic dimensions. For example, in the municipalities of Santos, Cubatão, and Paulínia, the number of cases reached an equivalent of 2.5% of the population in less than 140 days from the first diagnosis in the state, whereas in Mongaguá, Pitangueiras, and Santa Bárbara do Oeste, this level was reached after 450 days^13^.

Demographic density was the ecological factor most associated with mortality from COVID-19. More populated areas imply more intense social interactions, economic activities, mass transportation, vertical dwellings, and marginalized communities. These elements favor the progression of respiratory infections such as COVID-19. Likewise, the isolation of the most vulnerable groups is more difficult in these circumstances, explaining the higher mortality^14^.

The social inequality of municipalities, assessed by the Gini index, proved to be another relevant factor. In this scenario, the pandemic-imposed pressure on pre-existing vulnerabilities related to poor socioeconomic, educational, and health conditions in marginalized groups, in addition to the greater presence of comorbidities, which made them more susceptible to contagion and unfavorable outcomes. Added to this are reduced access to health services, overcrowded housing, difficulty in adhering to preventive measures of social distancing, and job insecurity^15^.

Municipalities with lower income per capita, as measured by HDI income, had higher mortality rates from COVID-19. Strong financial conditions for the population as a whole maximize adherence to individual care, resilience to social isolation, and collective efforts that the pandemic context requires^16^ ^,^ ^17^.

Lower life expectancy at birth, as measured by HDI longevity, was associated with higher municipal mortality, reflecting the aggregate role of different elements. Better healthcare, food safety, sanitation, public safety, and social well-being contribute to a simultaneous increase in longevity and capacity (individual and collective) to react to the contingencies imposed by the pandemic.

The highest latitudes of the municipalities were associated with higher mortality rates. Although this finding is repeated in studies of the incidence and mortality from respiratory infections worldwide^18^, its specific meaning is uncertain when considering low geospatial variation, such as that contained in the state of São Paulo. The possibility due to geographic interaction with neighboring states cannot be excluded.The behavior of the pandemic comprises the interaction of a complex system of factors, such as the pathogenicity of the virus, characteristics of the host, the way of transmission, hygienic and preventive measures, and the previous status of the pandemic. No study design can systematically approach all these factors simultaneously. Notably, ecological studies based on public records have operational inaccuracies. However, they are appropriate designs for evaluating health policies when exposure and outcomes are intrinsically dependent on collectivity.

These results cannot be extrapolated for other municipalities or for later stages of the pandemic, such as the status of the pandemic (e.g., daily new cases), healthcare support (e.g., shortage of ICU beds, oxygen supply), political management of the crisis, rate of vaccination, effective social distancing, and population access to timely and sensitive diagnosis, as these are situational determinants that influence the course of the pandemic. Finally, other ecologic factors, such as political corruption, financing management, ostensive epidemiological vigilance, and population leadership, affected the outcomes of each municipality.

In conclusion, elements linked to the host, virulence, and social determinants of health interact to shape population outcomes in COVID-19. The variation in mortality among the most populous municipalities in the state of São Paulo is influenced by demographic, geographic, economic, and social factors.
